# Dr. George W. Bowler

**Published:** 1884-07

**Authors:** 


Dr. George W. Bowler.—The readers of the Journal who have so often read the interesting articles from the pen of Dr. Bowler, will hear with regret the sad intelligence of his death, which took place at his home in Cincinnati on the 31st of May.

Dr. Bowler, who was 54 years of age at the time of his death, was a highly esteemed member of the profession.

